# Development of pathological skin models: from conventional techniques to 3D bioprinting

**DOI:** 10.3389/fbioe.2025.1538774

**Published:** 2025-09-01

**Authors:** Maïté Rielland, Françoise Bernerd, Marie Camman, Xuezhu Tan, Nathalie Seyler

**Affiliations:** ^1^ L’Oréal Research and Innovation, Aulnay-sous-Bois, France; ^2^ Episkin, Lyon, France

**Keywords:** human skin, pathological skin, bioprinting, reconstructed skin, bioengineering

## Abstract

Reconstructed human skin models were first developed in the 1970s. Since then, they have played a pivotal role in dermatological research, significantly advanced our understanding of skin biology, and brought huge insights into dermatological pathologies. Many conventional pathological skin models exist covering a wide range of diseases including melanomas, psoriasis, atopic dermatitis, genetic disorders, and wound healing conditions. However, conventional skin models remain limited by technical constraints which prevent complete replication of the spatial organization (heterogeneities, microenvironment) of skin diseases. Bioprinting has emerged as a powerful technology with the potential to overcome some of these limitations. By enabling precise control over the spatial organization of multiple cell types within a tailored extracellular matrix, bioprinting facilitates the creation of complex, three-dimensional skin models that closely mimic the architecture and function of human skin. This review initially explores the current landscape of conventional reconstructed pathological skin models. Bioprinting techniques, bioink considerations, and their roles in creating complex skin models are discussed. It then highlights the benefits of bioprinting for tissue microenvironment replication, architectural fidelity, and integration of multiple cell types in pathological skin models. In terms of healthy skin models, three-dimensional bioprinting is already revolutionizing personalized medicine, automating model production, and supporting translational research and therapeutic and cosmetic screening. It also represents a transformative approach for developing advanced pathological skin models despite the remaining technical and regulatory challenges.

## 1 Introduction

Human skin is the largest organ in the body and forms the first line of defense against chemical, physical, and pathogenic agents. To fulfil this defensive role, it has a complex multi-layered structure. The epidermis is the uppermost layer and is extremely cellular, containing mostly keratinocytes, but also Langerhans cells and melanocytes. Under the epidermis is the dermis, which is mostly acellular, containing extracellular matrix (ECM), fibroblasts, vessels, and annexes (pilosebaceous unit, glands), and then the hypodermis containing adipocytes and ECM (Dréno, 2009; Kanitakis, 2002; Hofmann et al., 2023a). The cells within these layers have different embryonic origins and functions. For example, keratinocytes and fibroblasts have structural and barrier functions, melanocytes produce melanin, Langerhans cells provide immunity, endothelial cells are needed for vascularization, and gland structures contain sebaceous and sudoriferous cells. Additionally, skin organization can vary between anatomical sites and individuals, and during pathological processes. Factors including external aggressors (UV exposure, pollution), lifestyle, ageing, and disease cause the skin structure to continually change (Wong et al., 2016; Oh et al., 2014; Fernandez-Flores, 2015).

The development of bioengineered skin began in 1977, with reconstructed epidermis *in vitro*, and was followed in 1981 by a full-thickness model comprising an epidermis and a dermal equivalent, initially developed for wound healing and skin grafts (Bell et al., 1981). Bioengineered skin substitutes have since been used for research and development to improve understanding and for modelling the physiology of normal or pathological skin (Ali et al., 2015; Daikuara et al., 2021; Albanna et al., 2019; Chouhan et al., 2019). They have also been validated for testing the potential efficacy and safety of drugs and cosmetic products (Lelièvre et al., 2007; Wever et al., 2015).

However, conventional skin models cannot completely replicate the complex cellular composition and microenvironment of native human skin. Three-dimensional bioprinting represents a transformative approach that has been used to develop healthy skin models. However, developments are still needed using this technique to produce advanced pathological skin models that more closely mimic *in vivo* conditions.

The objective of this review is to outline the different skin models, focusing particularly on pathological models, created using conventional tissue engineering techniques and to examine how three-dimensional bioprinting could improve *in vitro* pathological skin modelling.

## 2 Conventional tissue engineered skin models

### 2.1 Healthy skin models

**FIGURE 1:**
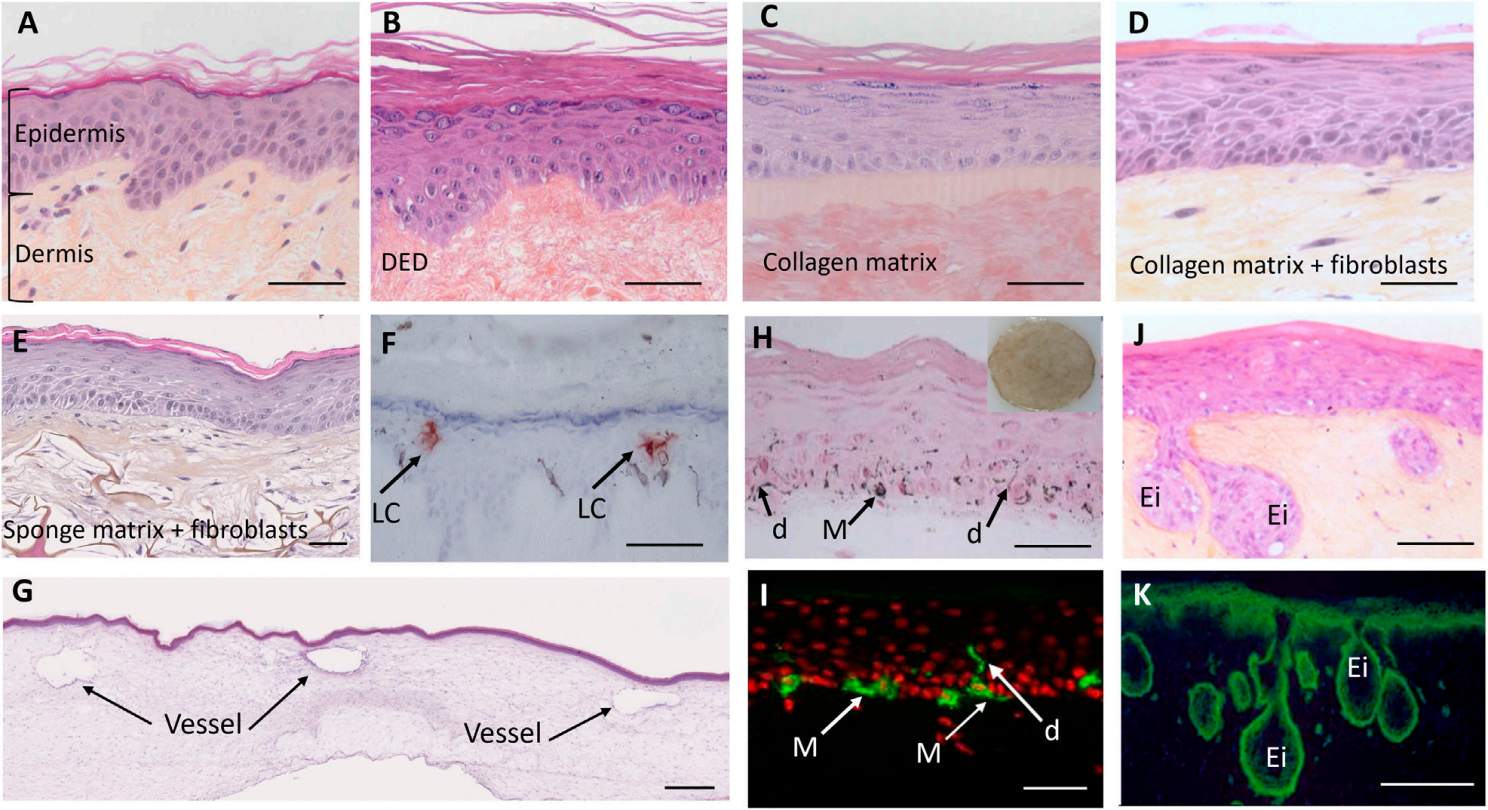
Morphological illustrations of human skin and 3D models created using conventional culture methods. **(A)** normal human skin (breast). **(B)** reconstructed human epidermis (RHE) cultured on a dead de-epidermized dermis (DED) support. **(C)** RHE cultured on an acellular collagen matrix Episkin™ model, **(D)** full thickness reconstructed skin model comprising an epidermis cultured on a living dermal equivalent composed of fibroblast populated contracted collagen matrix (lattice model). **(E)** full thickness reconstructed skin comprising an epidermis and a living scaffold dermal equivalent composed of a sponge matrix populated by dermal fibroblasts and left in culture conditions for 3 weeks to allow the fibroblasts to produce their own dermal matrix. **(F)** RHE containing Langerhans cells (LC) on a DED support. **(G)** vascularized full thickness reconstructed skin comprising an epidermis cultured on a dermal equivalent with endothelial cells, reproducing small vessels with their lumen. **(H,I)** full thickness reconstructed skin (lattice model) comprising a pigmented epidermis with melanocytes (M) able to produce melanin pigments (dark points) that are transferred to keratinocytes through the dendrites (d). **(J,K)** pathological Xeroderma pigmentosum group C (XP-C) full thickness reconstructed skin, comprising an epidermis obtained using XP-C Keratinocytes and a living dermal equivalent populated with XP-C Fibroblasts. The model reveals interactions between both compartments leading to the formation of epidermal invaginations (Ei) within the dermal compartment highly reminiscent of early stages of neoplasia. **(A–J)** histological sections stained with hematoxylin eosin saffron; **(F)** frozen skin section with anti-Langerin antibody immunostaining to reveal Langerhans cells (brown), **(H)** histological section after Fontana Masson staining to reveal melanin pigments, **(I)** frozen skin section with anti-tyrosinase-related protein (TRP-1) antibody immunostaining, **(K)** frozen skin sections of Xeroderma pigmentosum patients with anti β1‐integrin antibody immunostaining (green). Ei: epidermal invaginations. Bars **(A–K)** = 50µm; bar **(G)** = 500 µm (Image source: L’Oréal Research and Innovation).

Historically, the first *in vitro* 3D models were restricted to the epidermis, based on the keratinocyte culture technique (Rheinwald and Green, 1975). These Reconstructed Human Epidermis (RHE) models use normal human keratinocyte cultures seeded on a support, such as a polycarbonate film or acellular collagen matrix, which is immersed in culture medium, and finally brought up to the air-liquid interface to trigger epidermal differentiation (stratum basale, stratum spinosum, stratum granulosum, stratum lucidum, and stratum corneum) (Hofmann et al., 2023a; Boehnke et al., 2007). This epidermis structure reproduces the physiological, biochemical, molecular, and morphological characteristics of native human epidermis (el-Ghalbzouri et al., 2002; Nakamura et al., 1990; Groeber et al., 2011) (Figures 1A–C).

In addition to grafting, RHE models have been extensively used to better understand the physiology of healthy skin and for safety testing of raw materials and products, especially for cosmetics, as an alternative to animal testing (Rheinwald and Green, 1975). Several models are now produced in an “industrial” process and commercialized. Three commercially available RHE models (EpiSkin^®^, SkinEthnic^®^ and Epiderm^®^) are now approved for substance hazard assessments (skin irritation and corrosion) (Hofmann et al., 2023a).

However, despite the varied uses of RHE, it has been shown that fibroblasts are essential for complete epidermal histogenesis (Boehnke et al., 2007; el-Ghalbzouri et al., 2002), revealing the importance of full-thickness skin models to reproduce native skin characteristics more accurately (Nakamura et al., 1990; Groeber et al., 2011). Full-thickness skin models are produced by preparing a fibroblast-populated dermal equivalent, for example, by seeding fibroblasts onto a scaffold and allowing them to produce their own ECM, and then seeding keratinocytes onto the dermal equivalent (Black et al., 2005; Weigel et al., 2022). As with RHEs, once the dermal equivalent is seeded with keratinocytes, the full-thickness models are immersed in culture medium then lifted to the air to trigger keratinocyte differentiation (Hofmann et al., 2023a; Sriram et al., 2015). Since the historical lattice model created by Bell and colleagues in 1981 (Bell et al., 1981) based on a contracted gel composed of native type I collagen and dermal fibroblasts (lattice model) (Asselineau et al., 1985), other substrates have been successfully used including hydrated collagen gel (Trent and Kirsner, 1998), fibrin-based gels (Mazlyzam et al., 2007; Montero et al., 2021), chitosan cross-linked collagen-glycosaminoglycan lattice (Saintigny et al., 1993; Boyce et al., 1990) and scaffolds (sponge matrix, for example,) populated with fibroblasts which produce their own matrix (Bioarray). Illustrative examples are shown in Figures 1D,E.

As with RHE, commercialized full-thickness models are also now available, such as Advanced Skin Test 2000^®^, CreativeBioArray Full Thickness model, MatTek EpiDerm FT™ or Phenion FT long life (Imran et al., 2024).

To achieve even more accurate physiological models and to assess other functionalities, additional cell types have been progressively introduced. Melanocytes, responsible for melanin synthesis, can be incorporated into RHE and full-thickness models (Régnier et al., 1999; Duval et al., 2012; Supp et al., 2020) to reproduce constitutive, sun-induced pigmentation or to test depigmenting agents. Langerhans cells, the epidermal antigen presenting cell, have also been introduced in the epidermis to address some epidermal immunologic aspects (Régnier et al., 1997). It is also possible to add immune cells, vessels, nerves, and adipose tissue (Hofmann et al., 2023a; Black et al., 1998; Salameh et al., 2021). Some examples are presented in Figures 1F–I.

There is extensive, existing literature on healthy skin models (Groeber et al., 2011; Imran et al., 2024; Suhail et al., 2019). However, as this review focuses on pathological models, this will not be covered in detail here.

### 2.2 Pathological skin models

Currently, the Nuremburg Code requires animal models for preclinical trials, but significant differences have been observed between the phenotypes displayed in humans and animal models (Shuster, 1997; Wall and Shani, 2008). This has made the use of 3D pathological skin models an increasingly attractive alternative (Stanton et al., 2022). Understanding the complex processes and interactions between the different cell types is also key to increasing understanding about disease pathophysiology.

Initially, to create pathological skin models, genetically modified cells or cells from patients were introduced into healthy skin models to reproduce the desired pathology. However, the microenvironment is a key parameter in disease development and pathological models have therefore often needed additional adaptation to ECM organization such as adding vascularization or immune components. Additionally, pathological model lifespan was a limiting factor in studying disease progression but, recent advances to improve complexity in healthy models have enabled pathological models to progress.

#### 2.2.1 Cancer

Skin cancers represent a huge percentage of cancers diagnosed each year with more than 9000 people being diagnosed with skin cancer every day in the U.S.A. (Siegel et al., 2022; American Academy of Dermatology Association). The major skin cancers are Non-Melanoma Skin Cancers (NMSC) including basal cell carcinomas (BCC) and squamous cell carcinomas (SCC) and melanomas. Although melanomas are less common than NMSC, they are more invasive.

Since cancers are caused by cellular mutations, researchers have tried to create skin cancer models by introducing genetically modified healthy cells or cells obtained from patients with cancer into healthy skin models. However, cancer progression is often due to interactions between cells and the microenvironment meaning complex pathways must be modelled as shown below.

##### 2.2.1.1 Melanoma

It has been speculated that disrupting melanocyte/keratinocyte homeostasis triggers melanoma cells to form from melanocytes entering a radial growth phase. A vertical phase then occurs causing the melanoma cells to penetrate the basement membrane and blood and lymph vessels forming metastases in different areas of the body (Bandarchi et al., 2010; Uong and Zon, 2010; Hsu et al., 2002). Interactions between the metastatic cells and surrounding tissue determine the clinical outcome. Melanoma skin substitutes (MSS) have been used to investigate these interactions (Eves et al., 2003), and confirm that melanoma cells in the radial growth phase are unable to cross the basement membrane but cells from the vertical growth phase can (Meier et al., 2000; Haridas et al., 2017).

MSS have also been used to investigate the interaction of treatments with melanoma cells (Grindon et al., 2006) and identify new signaling pathways leading to potential new treatments. An MSS was used to show that sorafenib combined with rapamycin can prevent melanoma cells from crossing into the dermal compartment (Folkman, 1990).

Additionally, the creation of an MSS containing blood vessels and lymphatic capillaries has improved research into melanoma pathogenesis (Bourland et al.). Since some types of melanoma can evade immune detection, developing MSSs capable of this would improve understanding of this process (Passarelli et al., 2017). Furthermore, using patient-derived melanoma cells may also help provide personalized effective treatments.

##### 2.2.1.2 NMSC: BCC and SCC

Both BCC and SCC carcinomas arise from mutated keratinocytes. The initial events have been linked to sun exposure, and UV-induced DNA lesion formation such as cyclobutene pyrimidine dimers (Brash et al., 1996). Impaired repair of these DNA lesions leads to specific mutations (UV signature) and tumor development.

Recapitulating the tumor microenvironment is one challenge faced when developing 3D carcinoma models. Several models have been proposed mostly based on the use of mutated SCC keratinocyte cell lines (Oudda et al., 2023).

However, due to the causal role of UV exposure in the development of skin cancers, efforts have been made to improve understanding of early related events such as DNA lesion formation and repair and UV-induced mutagenesis (Marionnet and Bernerd, 2016; Marionnet and Bernerd, 2019). Other approaches have attempted to use cells from diseased patients with a high predisposition to skin cancers (see Genodermatosis section).

The major limitations of skin cancer models include obtaining adequate amounts of cells with the required mutation and creating the correct microenvironment with vascularization and immune cells. Furthermore, the limited lifespan of skin cancer models makes it very difficult to study cancer progression.

#### 2.2.2 Psoriasis

Due to the polygenic, multifactorial nature of psoriasis, constructing an *in vitro* model which mimics all these features is challenging (Niehues and van den Bogaard, 2018). Different approaches have been tested, from using psoriatic cells to models incorporating various cell types to model cellular interactions. Using full-thickness models is beneficial as fibroblasts have been shown to participate in psoriasis pathogenesis (Angiolilli et al., 2022; Berroth et al., 2013; Gęgotek et al., 2020). Due to the role of inflammatory components, some models add psoriasis-associated cytokines to healthy keratinocytes causing them to behave like psoriatic keratinocytes (Tjabringa et al., 2008) producing epidermal acanthosis and parakeratosis (Boniface et al., 2005; Nograles et al., 2008; Sa et al., 2007). However, in these models, keratinocyte hypertrophy caused acanthosis rather than hyperproliferation meaning the hyperproliferative epidermis, a hallmark lesion of psoriasis, was not mimicked accurately. However, a psoriasis model has been created recently featuring hyperproliferation, parakeratosis, impaired cell attachment, inflammation, and differentiation defects (Morgner et al., 2023). To overcome the limitations of historical psoriatic models, the authors (Boniface et al., 2005) used primary keratinocytes seeded on a fibroblast-derived dermal matrix and combined them with recombinant Th1 and Th2 cytokine stimulation to mimic psoriatic T-cell-signaling. One of the advantages of this model may rely on the higher diffusion properties of the substrate.

Since psoriasis has known pathology-causing genes, therapeutic gene silencing is a potential therapeutic tool through RNA interference. *In vitro* psoriasis models were used to test the therapeutic efficacy of an RNAi treatment which was shown to successfully silence four target genes, each of which play a different role in psoriasis (Desmet et al., 2018).

T cells have also been incorporated into psoriasis models to study the interaction between keratinocytes and immune cells (van den Bogaard et al., 2014). However, these models lack the psoriatic morphological hallmarks. Due to the innate challenges, very few models include other immune cells which are also involved in psoriasis pathogenesis and progression. It would also be beneficial to include dendritic cells, leucocytes, and blood vessels to help predict *in vivo* treatment responses (Stanton et al., 2022).

#### 2.2.3 Atopic dermatitis

Atopic dermatitis (AD) is an autoimmune skin disease characterized by a combination of increased immune cell infiltration, T cell-mediated inflammation, epidermal barrier disruption, and filaggrin downregulation (Jang et al., 2023; Bieber, 2008). To mimic this complex disease, AD models must reflect the TH2-mediated inflammatory conditions as this is a key inducer of AD pathogenesis. Activated T cells have been used in models (Engelhart et al., 2005; Wallmeyer et al., 2017) but Th2 cell behaviors and characteristics change during the immune response making reproducibility impossible. Some researchers are using a cytokine mixture instead, the best combination of which to correctly mimic AD is still being investigated (De Vuyst et al., 2018; Danso et al., 2014; Rouaud-Tinguely et al., 2015; Yuki et al., 2016). However, these methods cannot accurately mimic AD pathophysiology or the complex inflammatory cascade which is a central pathogenic mechanism (Jang et al., 2023).

Some strategies involve genetic mutation or suppression, particularly FLG-encoding gene mutation since filaggrin depletion is a key AD characteristic (Akiyama, 2010; Elias et al., 2017). However, despite mimicking barrier malfunction, these models do not display the inflammatory cascade (Pendaries et al., 2014; Pendaries et al., 2015; Küchler et al., 2011; van Drongelen et al., 2013).

The impact of AD on the skin microbiome is of increasing interest. Increased levels and colonization of *S. aureus* creating microbial imbalance is a well-known aspect of AD so researchers have begun to introduce it into *in vitro* AD models (Kim et al., 2019; Abeck and Mempel, 1998; Choi et al., 2018). However, the skin microbiome is hugely diverse, complex and specific to each individual meaning colonization with *Staphylococcus aureus* alone cannot adequately mimic the *in vivo* situation (Jang et al., 2023). This area requires further investigation.

Another improvement could be to use patient-derived AD and immune cells. This could improve understanding about patient-specific responses to different treatments (Stanton et al., 2022).

#### 2.2.4 Genetic skin diseases (genodermatosis)

The most common method used to create genetic skin disease models is to introduce cells from patients or genetically modified cells directly into healthy skin models to reproduce the etiologic defect.

##### 2.2.4.1 Ichthyosis

Ichthyosis vulgaris is the most common form and is characterized by filaggrin null mutations causing a depletion of filaggrin which plays a role in stratum corneum integrity (Leman et al., 2019). Using RNA interference technology to silence filaggrin within keratinocytes is one method for studying filaggrin knockdown (Mildner et al., 2010; Mildner et al., 2006). Genetic knockout has also been used to study harlequin ichthyosis, a more severe form (Enjalbert et al., 2020). This has provided valuable information but there are genes implicated in this disease which are yet to be investigated using *in vitro* models (Stanton et al., 2022).

##### 2.2.4.2 Epidermolysis bullosa

Epidermolysis bullosa (EBA) is an inherited skin disease which causes skin fragility and recurrent blisters (McGrath, 2015; Fine, 2010). EBA is the non-inherited, autoimmune form caused by autoantibodies to type VII collagen and causes mucocutaneous blisters (Kasperkiewicz et al., 2016). Models produced to study these conditions (Lincoln et al., 2018; Akasaka et al., 2021; Rossi et al., 2015) provide an adequate representation of the disease but fail to demonstrate how it affects adnexa and skin layers. Model efficacy would be improved with a properly constructed dermoepidermal junction (Fine, 2010). Using immune-competent models to study EBA would enable the role of the immune system to be studied (Stanton et al., 2022).

Genetic EB with mutations in type VII collagen gene or Lam 332 have also been tested for 3D reconstruction with success (Spirito et al., 2006; Gache et al., 2004). In these 2 cases, approaches to achieve genetic correction of keratinocytes through transduction of normal genes have been shown. These studies are rare examples demonstrating a translational process from *in vitro* to *in vivo* therapy using the skin model approach. However, the *in vivo* assays were performed in dogs or mice, not human clinical trials.

##### 2.2.4.3 Xeroderma pigmentosum

Xeroderma pigmentosum (XP) is a rare recessive genetic disease, characterized by defective UV-induced DNA lesion repair. Seven groups of XP (XP-A to XP-G) and one variant form (XP-V) have been identified depending on the mutated gene (Sarasin, 1999). The major clinical characteristic is sensitivity to solar light, mostly UV rays, leading to a higher incidence of skin cancers and premature signs of skin ageing occurring during childhood. Due to the low incidence of the disease, very few *in situ* studies have been performed. Using cell strains (keratinocytes and fibroblasts) from XP-C donors, reconstructed skin with defective DNA repair has been created which can reproduce the functional DNA repair defect after UV exposure (Bernerd et al., 2001). Additionally, a full-thickness 3D XP skin model revealed abnormalities in the dermal compartment, notably a photoaging-like phenotype of XP-C fibroblasts (Fréchet et al., 2008). The presence of XP-C fibroblasts was shown to induce epidermal invaginations, closely resembling early neoplastic events, thus highlighting specific interactions between the 2 cell types in the tumor progression process (Figures 1J,K). This illustrates how pathological models can be used to explore new mechanistic features of a disease. These models have also been used as a preclinical model of genetic correction, aimed at restoring the DNA repair function by reintroducing a WT XP-C gene into diseased cells. The transduced XP-C keratinocytes showed recovery of DNA repair following UV exposure (Warrick et al., 2012) but, as with EBA, these trials were only performed in mice.

##### 2.2.4.4 Nevoid basal cell carcinoma/gorlin-goltz syndrome

Nevoid Basal Cell Carcinoma/Gorlin-Goltz Syndrome (NBCCS) is an autosomal dominant inherited disorder characterized by a very high predisposition to BCC (Gorlin, 1995). A huge majority of sporadic BCCs bear somatic mutations in the PATCHED1 tumor suppressor gene which encodes the receptor for the Sonic Hedgehog morphogen (SHH) (Soufir et al., 2006). SHH transduced keratinocytes were used to create human transgenic skin which displays the abnormal specific histologic features seen in BCCs, including downgrowth of epithelial buds into the dermis, basal cell palisading, and epidermal separation from the underlying dermis (Fan et al., 1997). By using skin cells directly derived from patients with NBCCS, 3D skin reconstructs could be generated, early biomarkers of epithelial neoplasia development recapitulated, and new insights into the role of dermal fibroblasts in epidermal carcinogenesis revealed (Brellier et al., 2008; Gache et al., 2015).

Skin models have also been made to study ankyloblepharon-ectodermal defects-cleft lip/palate (AEC) syndrome characterized by *TP63* gene mutations leading to aberrant keratinocyte differentiation (Koch et al., 2014; Zarnegar et al., 2012), Kearns-Sayre syndrome which is a mitochondrial disease associated with large amounts of UV-induced mitochondrial deletion and premature aging (Majora et al., 2009), and type I autosomal recessive Cutis Laxa, a rare genetic skin disease associated with premature signs of dermal skin ageing and Fibulin five gene mutation (Claus et al., 2008).

These data demonstrate that single-gene defect genodermatoses represent the easiest pathologies to mimic using appropriate genetic manipulation of the cells of interest. They have been extensively used for research purposes, helping to increase knowledge, especially for rare genetic diseases. Clinical translation towards therapeutic approaches remains challenging due to regulatory aspects, use of vectors adequate for human long-term safety, and obtaining patient cells for autografts.

#### 2.2.5 Connective tissue skin diseases and wound healing models

##### 2.2.5.1 Scleroderma

This disease is characterized by connective tissue remodeling and has hereditary and acquired factors but is categorized as autoimmune (Abraham and Varga, 2005; Singh D. et al., 2019). Plasmacytoid dendritic cells play a central role in fibrosis development (Silva et al., 2023). One model containing dendritic cells revealed that inhibiting them could be a potential therapeutic avenue (Hunzelmann and Krieg, 2010). However, current models lack vasculature and immune system components, the inclusion of which would improve their relevance (Stanton et al., 2022).

##### 2.2.5.2 Normal and impaired wound healing

Normal wound healing involves a four-stage cascade of events: hemostasis, inflammation, granulation tissue formation, and scar formation with a balance between scar formation and remodeling (Groeber et al., 2011; Weiss, 1989; Martin and Leibovich, 2005). However, pathological responses to wounds can produce chronic ulcers, fibrosis or excessive scarring (Diegelmann and Evans, 2004).

2D *in vitro* models can be created by growing keratinocytes or fibroblasts into a monolayer which is then wounded (Liang et al., 2007; Pinto et al., 2016; Kramer et al., 2013). However, manual scratching of the monolayer is normally uneven, leading to potentially biased results and a lack of reproducibility (Kramer et al., 2013; Stamm et al., 2016). This can be overcome by using electric cell-substrate impedance sensing (Giaever and Keese, 1991) which improves reproducibility and provides real-time measurements but is expensive (Pflüger et al., 2013; Hundsberger et al., 2017; Hung et al., 2022; Ramasamy et al., 2014).

However, 2D models often only address one biological end point such as cell migration, fail to accurately reflect physiological conditions, and cell appearance and characteristics differ significantly from *in vivo* skin making it difficult to predict *in vivo* responses (Sun et al., 2006; Bhadriraju and Chen, 2002; Schreier et al., 1993). For this reason, 3D models have been extensively investigated (Niehues et al.; Garlick, 2007; Egles et al., 2010; Deshayes et al., 2018). These enable analysis of re-epithelization, cell interactions, and morphological changes including basement membrane reconstruction, and the structure and composition of neosynthesized ECM (Sun et al., 2006; Langhans, 2018; Iyer et al., 2018).

Chronic wounds are wounds which remain in a self-perpetuating inflammatory stage (Graves et al., 2022; Zhao et al., 2016) and do not heal within weeks or even months (Graves et al., 2022). One chronic wound model used fibroblasts from a diabetic foot ulcer and had key chronic wound features such as delayed re-epithelization, decreased revascularization, and keratinocyte hyperproliferation (Maione et al., 2015). However, to reliably predict the efficacy of new treatments, models representing the causes of chronic wounds are required (Hofmann et al., 2023b).

Excessive scarring (keloid and hypertrophic scars) is another potential result of pathological wound healing caused by excessive ECM deposition and prolonged proliferation of granulation tissue (Finnson et al., 2013; Mokos et al., 2017). Initial 3D keloid models used fibroblasts from the disease with a layer of normal epidermal keratinocytes (Butler et al., 2008). A refined version was produced with a dermal model made using fibroblasts from the keloid center and periphery, and healthy skin (Suttho et al., 2017) but no epidermis was created. Immunocompetent keloid models have also been made using CD14^+^ monocytic cells (Limandjaja et al., 2019). These models are being used to test potential treatments such as 5-fluorouracil as a monotherapy (Hietanen et al., 2019) or in combination with glucocorticoids (Darougheh et al., 2009; Apikian and Goodman, 2004), and radiation (Son et al., 2020).

Hypertrophic scars are normally a complication following extensive skin trauma or severe burns. Unlike keloids, they do not extend beyond the injured area but are elevated, uneven, painful, rigid, pruritic, and tend to improve over time (Eming et al., 2014). Myofibroblasts have been shown to persist in these scars contributing to the phenotype (Lee et al., 2019; Moulin et al., 2004). Deep dermal fibroblasts (Varkey et al., 2011), cutaneous adipose mesenchymal stem cells (van den Broek et al., 2012) and stress-activated fibroblasts (Derderian et al., 2005) have all been used in 3D models resulting in hypertrophic scar characteristics. Using fibroblasts from hypertrophic scars in 3D models produced models which accurately mimicked the *in vivo* characteristics (Phan et al., 2003; Linge et al., 2005; Younai et al., 1994). However, the mechanisms causing hypertrophic scars remain elusive and research into this area is still essential (Mokos et al., 2017). Incorporating vasculature and immune cells would also be beneficial.

#### 2.2.6 Infectious skin diseases

*Candida albicans* is a fungus which forms part of the epidermal and mucosal microbiome but, in immunocompromised people, can cause serious disease (Mishra et al., 2007). Oral and epithelial models have been infected with *C. albicans* (Schaller et al., 1999; Schaller et al., 2000; Green et al., 2004) and enabled researchers to study the pathogenicity, virulence, inflammatory response (Schaller et al., 2002), adhesion and penetration (Dieterich et al., 2002).

Three-dimensional *in vitro* skin models have also been used to study infectious viral skin diseases including Herpes Simplex Virus (HSV) (Visalli et al., 1997; Hukkanen et al., 1999; Syrjänen et al., 1996), papillomaviruses, and adenoviruses (Andrei et al., 2010).

## 3 The next steps

Despite these pathological 3D models having multiple uses and helping to advance the understanding about many different skin diseases and their treatments, there are still limitations and a need to build models which better mimic the *in vivo* situation. *In vitro* models made using conventional tissue engineering are size restricted, expertise dependent, and time consuming (Suhail et al., 2019). They are also made using a mix of cells that are deposited manually meaning they may not reproduce the pathology accurately, such as plaques or healthy/damaged areas. Areas requiring improvement include ease of production, creating a physiological microenvironment and ECM to mimic the healthy and pathological areas, and including specialized cells such as sebocytes, macrophages, and Merkel cells (Randall et al., 2018).

Bioprinting is a versatile, state-of-the-art biofabrication technique which could provide solutions to some of these limitations. Benefits of bioprinting include the ability to precisely control the tissue microenvironment and cell deposition, reproduce tissue complexity, create a 3D complex, multicomponent pathological skin model for different conditions, personalize skin models, and control, standardize, and automate production.

## 4 Bioprinting

Three-dimensional bioprinting is a versatile, advanced, and promising biofabrication technique to create complex skin models containing multiple components which better mimic native human skin. The spatial deposition enables skin heterogeneities to be reproduced.

There are different available bioprinting strategies which can be used to produce skin models (Sarkiri et al., 2019; Zhang et al., 2023). The most widely used and popular method is extrusion-based bioprinting (Zhang et al., 2023; Vijayavenkataraman et al., 2018). The viscous, cell-laden bioink is forced through a nozzle into a filament. The printhead moves building a 3D construct which is then cross-linked (Gu et al., 2020; Decante et al., 2021; Hull et al., 2022). This versatile and flexible technique ensures high cell viability and compatibility, and can be combined with other techniques including microfabrication, coaxial and multi-material bioprinting (Kim et al., 2017; Kim et al., 2021; Lian et al., 2022; Murphy and Atala, 2014). However, printing accuracy is not as good as other bioprinting techniques thus affecting cell spatial arrangement precision.

**FIGURE 2:**
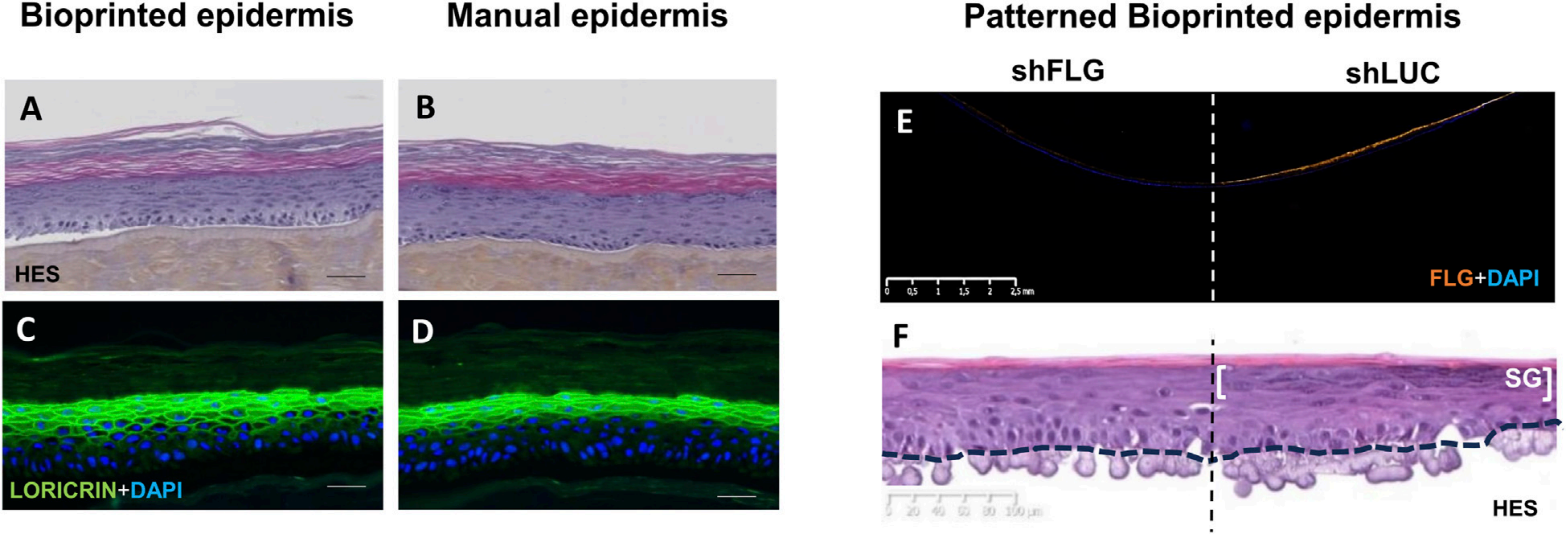
Morphological illustrations of bioprinted epidermis compared to manually obtained epidermis and patterned bioprinted epidermis. **(A,C)** bioprinted epidermis obtained using the inkjet method. **(B,D)** reconstructed epidermis created using manual seeding of the same human keratinocyte strain. **(A,B)** histological sections stained with (HES). **(C,D)** frozen skin section with Loricrin antibody immunostaining (granular layer staining in green) and DAPI nuclear counterstaining (blue). **(A–D)**: bar = 50 µm. **(E,F)** patterned reconstructed bioprinted epidermis with keratinocytes silenced for Filaggrin (shFLG) on one side and control keratinocytes (shLuc) on the other side. shFLG and shLuc keratinocytes are seeded on a single dermal support (acellular dermal matrix), the dotted line indicating the middle of the tissue which separates the two conditions. **(E)** frozen skin section containing the two conditions with filaggrin antibody immunostaining (orange) and DAPI nuclear counterstaining (blue). The absence of staining in the shFLG condition confirms filaggrin silencing in the epidermis. **(F)** histological section stained with (HES) revealing two distinct zones in the epidermis depending on the type of keratinocytes; one without keratohyalin granule formation corresponding to the shFLG condition and the other showing the presence of keratohyalin granules in the stratum granulosum (SG) corresponding to the control shLuc keratinocytes condition. Bars are indicated in the figure. (Image source: L’Oréal Research and Innovation).

Droplet-based bioprinting can be divided into inkjet bioprinting, electrohydrodynamic jet printing and laser-assisted bioprinting. Inkjet bioprinting uses continuous or on-demand bioink droplets to make skin models with high printing speed, resolution, and cell viability but requires low viscosity bioinks and has low cell concentration (Zhang et al., 2023; Gu et al., 2020; Gudapati et al., 2016; Kim and Kim, 2015) (Figure 2). With electrohydrodynamic jet printing, the bioink is pulled through a hole using an electric field. This technique has high precision and structural integrity but is expensive and printable materials are limited (Gudapati et al., 2016; Kim and Kim, 2015; He et al., 2020; Angkawinitwong et al., 2020; Haj-Ahmad et al., 2015; Sangnim et al., 2018; Lee S. et al., 2020). Laser-assisted bioprinting uses lasers to push bioink with high cell concentrations into a collector substrate. Like inkjet bioprinting, this technique also has high resolution and cell viability but is expensive and time-consuming (Albanna et al., 2019; Zhang et al., 2023; Gu et al., 2020; Kim and Kim, 2015; Guillemot et al., 2010).

Digital light processing bioprinting has a high printing speed and constructs stable structures with high resolution, structural integrity, and mechanical properties using precise lighting and photosensitive polymers (Zhang et al., 2023; Lee M. et al., 2020; Zhou F. et al., 2020). Other techniques include magnetic bioprinting, volumetric bioprinting and two photon bioprinting (Sörgel et al., 2023; Adine et al., 2018).

Innovative techniques are being used with 3D bioprinting to further improve skin model complexity. Cell-spheroids containing stem cells help skin equivalents better mimic native human skin though the stem cells must be precisely positioned and arranged to ensure the complex tissue is correctly created (Ong et al., 2018; Sun et al., 2022; Zhuang et al., 2018; Decarli et al., 2021; Kinney et al., 2014). Cell-spheroid laden bioprinting is particularly useful for skin appendages such as hair follicles (Zhang et al., 2021). Four-dimensional bioprinting (Douillet et al., 2022; Lee et al., 2021; Zhou W. et al., 2020), microfluidics-assisted extrusion (Ng and Yeong, 2019; Idaszek et al., 2019; Sutterby et al., 2020) and skin-on-a-chip technologies (Zoio and Oliva, 2022; Mori et al., 2017; Nahak et al., 2022) are also helping to advance skin tissue engineering.

## 5 Benefits of bioprinting for printing complex healthy and pathological skin models

### 5.1 The ability to precisely control the tissue microenvironment

With bioprinting, the location of different cells and ECM components can be precisely controlled allowing the specific tissue microenvironment of healthy and diseased tissue to be reproduced.

**FIGURE 3:**
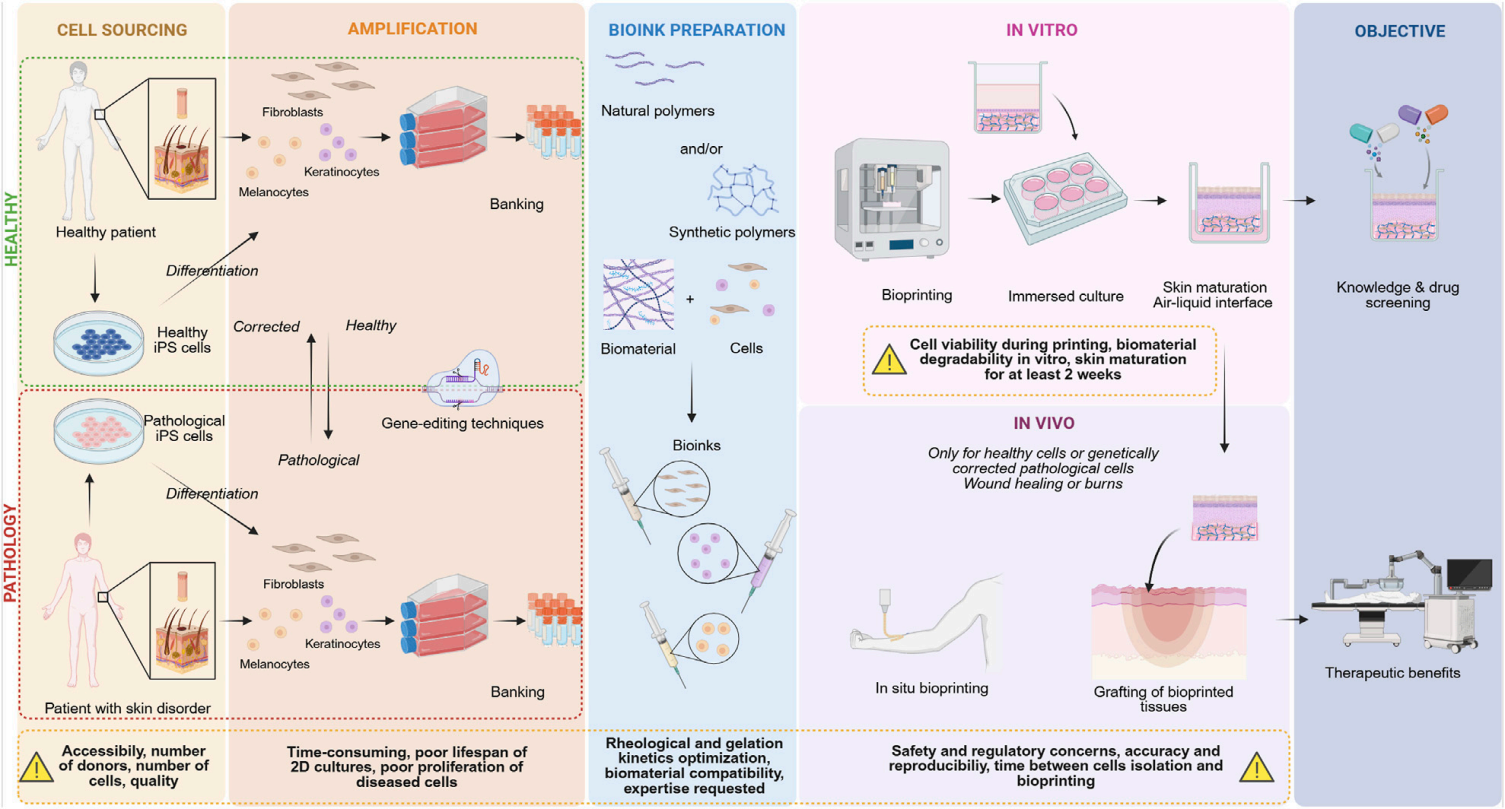
Bioprinting from bench to bedside and the limitations at each step of the process. Created in Biorender https://Biorender.com/wmr2gar.

Developing the right bioink is the key in bioprinted skin models and multiple assays are required to find the suitable parameters. When bioprinting a pathological skin model, an extensive understanding of the pathology in patients, how it affects the skin, and disturbs the cell microenvironment is essential. This knowledge is used to guide what biomaterial(s) will be used in the bioink. Pathological or healthy cells are added to the biomaterial(s) to create the desired bioink(s) which mimics either pathological or healthy skin (Figure 3). The bioink can reproduce the desired microenvironment (including alignment, porosity, mechanical properties). For example, if the skin disease causes fibrosis, the bioink will be developed to be stiff but, if the disease causes poor vascularization, the bioink must be less porous to reduce cell access to nutrients. Additionally, the bioink is chosen according to the bioprinting technique, the cell type, and the duration the bioink needs to remain stable in culture conditions. The biomaterial, photoinitiator and/or additional cross linker must also be compatible with the cells. Even if the bioink is compatible with the cells, the bioprinting process might be harmful to them. For example, stress caused by extrusion, light induced reactive oxygen species, chemical compounds, temperature, and humidity. Bioink printability must therefore be checked and optimized to maximize cell survival. Furthermore, bioinks need to maintain their shape after printing and crosslinking with limited swelling or contraction, unless this is required for the model. Therefore, bioink aging must also be studied to assess cell interactions with the ink.

Natural hydrogels are biocompatible, degradable enabling cell migration and proliferation, have high water content and low immunogenicity, and can contain amino acids which promote cell growth, maturation, and adhesion (Zhang et al., 2023; Kang et al., 2022a). Furthermore, substances such as amniotic epithelial cells (AEC) and Wharton’s jelly derived mesenchymal stem cells (WJMSC) can be incorporated into natural hydrogels such as alginate or gelatine. When incorporated in this way, they have been shown to increase expression of genes involved in wound healing, epidermis development, cell adhesion, cell junction assembly and vascular endothelial growth factor (Liu et al., 2019). However, natural hydrogels have poor mechanical properties.

Synthetic hydrogels have better mechanical properties that can be similar to that of native human skin (Kang et al., 2022a). Additionally, synthetic hydrogels can improve the stiffness or elasticity of natural hydrogels and build versatile and adaptable scaffolds (Kang et al., 2022a; Miguel et al., 2019). Combining bioink materials can help create bioinks with good 3D printability, biocompatibility and biofunctionality. In fact, bioinks help 3D bioprinted skin to have better long-term maintenance of shape and size compared with manually constructed skin equivalents (Vijayavenkataraman et al., 2018).

The complex microenvironment associated with skin diseases means the choice of biomaterials is even more important for pathological skin models (Nyström and Bruckner-Tuderman, 2019; Wells et al., 2016). One promising choice is decellularized extracellular matrix (dECM) bioinks (Dzobo et al., 2019). These have residual cells removed leaving just the ECM components and biomolecules such as growth factors, cell adhesion proteins and glycosaminoglycans which are found in native ECM (Kang et al., 2022a). This can result in a diverse range of biological, chemical, and structural cues which are useful for producing pathological skin models as they impact cellular behavior and mechanical properties (Kim et al., 2020; Choudhury et al., 2018).

It is also possible to have ECM which is completely neosynthesized by the cells in bioprinted or 3D printed scaffolds, for example, in the bilayer model (Girard et al., 2024) and solution electrospinning (SES) models (Black et al., 2005; Weigel et al., 2022; Dos Santos et al., 2015).

However, the pursuit of perfect printability is compromising the biocompatibility and mechanical strength of bioinks since they need to be viscous enough to be printed and to have high shape fidelity. To overcome this, hydrogels with good biocompatibility but poor strength, such as collagen, could be mixed with less biocompatible but stronger hydrogels such as gelatine and sodium alginate (Marques et al., 2019).

Scaffold printing patterns can be changed to alter dermal collagen fiber organization. For example, it has been shown that using a straight melt electrowriting (MEW) fiber model will produce homogeneously organized fibrillar collagen whereas a wavy design produces heterogeneously arranged collagen (Girard et al., 2024). It is also possible to create models with similar mechanical properties to *ex vivo* skin such as the first advanced bilayer scaffold/membrane model produced using melt electrowriting (MEW) and solution electrospinning (SES) (Girard et al., 2024).

Bioprinted scaffolds produce porous structures which enable cells to migrate, proliferate, and grow (Sarkiri et al., 2019) and can improve nutrient and metabolite circulation (Singh YP. et al., 2019; Liu et al., 2016). These scaffolds also give biological and biomechanical signals for cell survival (Daikuara et al., 2021). For example, biomaterials need to have cellular adhesion sites and cellular extension, proliferation, and adhesion can be promoted through good degradability (Yao et al., 2019). Furthermore, intracellular mechanosensitive proteins sense environmental biomechanical changes which influence cellular processes including fibrosis and differentiation (Chaudhuri et al., 2020). Cells and the microenvironment can also be influenced by mechanical forces and changes to biological materials resulting from cellular behavior. Biochemical signals from neighboring cells or the microenvironment also influence cell behavior. These signals can potentially be mimicked by adding substances such as platelet rich plasma, placental mesenchymal stem cells or dECM to the scaffold bioinks which in turn can impact the microenvironment of the skin equivalent (Zhang et al., 2023).

### 5.2 The ability to reproduce tissue complexity

Many diseases involve complex changes in tissue architecture. Bioprinting can allow you to reproduce these changes with high fidelity, making models more realistic and useful for studying different skin diseases. Biomaterials, cells, and growth factors can be printed simultaneously helping to produce more complex engineered skin models (Sarkiri et al., 2019; Vijayavenkataraman et al., 2018). In fact, the spatial positioning accuracy obtained with bioprinting enables keratinocyte heterogeneity to be reproduced in vitro models. This is successfully demonstrated with an innovative patterned bioprinting technique which created a stable heterogeneous epidermal model (Madiedo-Podvrsan et al., 2021) with the two-halves consisting of a different keratinocyte subpopulation (Figures 2E,F). The technique enabled precise and controlled bioprinting of separate keratinocyte populations to construct accurate patterns. These keratinocytes remained in their localization during culture producing a complex, compartmentalized model with improved stability and viability compared with previous patterning techniques (Guillotin et al., 2010; Ng et al., 2018). Two designs were also tested. The semi-circle design can easily be divided into two-halves to allow two different keratinocyte populations in one model to be evaluated. However, printing in concentric rings produces a more accurate model of heterogeneous skin areas.

3D bioprinting can also incorporate cells and molecules to encourage vascularization, innervation and pigmentation (Cui et al., 2012). Accurate cell deposition has enabled a full thickness pigmented skin model to be produced with the melanocytes positioned to mimic pigmented blemishes (Ng et al., 2018).

Vascularization is important as the blood vessels not only provide nutrients but regulate hair follicle stem cell activation (Li et al., 2019), promote sweat gland morphogenesis and regeneration (Yuan et al., 2023), and contribute to skin disease development, particular tumors, and autoimmune conditions (Rodrigues et al., 2019; Moreira and Marques, 2022). The most common method to produce vessels is by embedding a printed structure into the scaffold which is then dissolved to form a hollow cylinder. Vascular endothelial cells are then perfused and adhere to the channel creating vessels (Shao et al., 2020). Coaxial bioprinting is also able to construct vascularized tissue (Mohan et al., 2022; Liang et al., 2020). However, these methods only produce a single cell layered tube which does not accurately mimic the complex vascular network found within skin. A recent 3D bioprinted full thickness skin model used bioink containing human placental pericytes, endothelial cells, fibroblasts to form the dermis and a keratinocyte-containing bioink to create the epidermis. The endothelial cells and pericytes self-assemble into an interconnected microvascular network. When used as a graft on a mouse, the mouse’s microvessels inosculated with the microvasculature of the graft and was properly perfused 4 weeks after implantation (Baltazar et al., 2020). However, models recreating vascular alterations specific to different skin conditions are still a major challenge. Furthermore, model thickness is limited to 200 µm if no vascularization is included due to poor nutrient diffusion.

Appendages such as hair follicles, sweat glands and sebaceous glands are also an important part of skin and add to its complex structure. Building skin models containing appendages is important since without them it is impossible to test medical treatments for conditions such as folliculitis or alopecia. Some progress is being made in this area. Hair follicle spheroids were seeded into a 3D-bioprinted model containing sweat glands to construct a model with both hair follicles and sweat glands (Zhang et al., 2021). Other techniques include using gelatine methacryloyl/hyaluronic acid methacryloyl (GelMA/HAMA) bioink laden with normal human dermal fibroblasts and hair follicle dermal papilla cells which produced hair pore structures (Kang et al., 2022b). Stem cells have also been used to seed models that have the correct microenvironment to encourage these cells to differentiate into functional sweat or sebaceous glands (Yao et al., 2020; Chen et al., 2020).

### 5.3 The ability to create a 3D complex pathological skin model for different skin conditions

Creating multicellular tissue models with precisely arranged cells enables the interaction between healthy and pathological cells or between different pathological cell types to be studied. 3D bioprinted pathological skin models have been constructed for conditions including diabetic skin, atopic dermatitis, hypertrophic scars, and melanomas.

A 3D bioprinted diabetic skin model has been produced using diabetic dermal fibroblasts and diseased human subcutaneous preadipocyte cells to produce the dermal compartment. Normal human keratinocytes interacting with this diabetic dermis produced a diabetic epidermis by stimulating intercellular crosstalk between the dermis and epidermis as occurs in native human skin. This model showed insulin resistance, adipocyte hypertrophy, slow re-epithelialization, and inflammatory reactions (Kim et al., 2021).

To build an atopic dermatitis (AD) model, induced pluripotent stem cell-derived endothelial cells, pericytes, fibroblasts and keratinocytes were used to create a vascularized full-thickness model with T_h_2 cytokine IL-4 added to the culture medium. This cytokine is involved in AD pathophysiology. The resulting AD model had increased pro-inflammatory cytokine levels, early and terminal expression of differentiation proteins, hyperplasia and spongiosis which are all hallmarks of AD (Liu et al., 2020). Patterned bioprinting has been successfully used to produce a model to study the edges of AD lesions. For this, keratinocytes with either a normal or low filaggrin expression were used. This resulted in a stable heterogeneous model with each half expressing the phenotypic characteristics of the keratinocytes used such as a characteristic decrease in keratohyalin granules (Madiedo-Podvrsan et al., 2021) (Figures 2E,F).

Scar dECM enhanced alginate-gelatine hydrogel containing pre-cultured patient-derived fibroblast aggregates (preformed cellular aggregates) was used to construct a hypertrophic scar model with functional scar tissue self-organization and gene and protein expression characteristic of early-stage hypertrophic scars (Bin et al., 2022).

Melanoma models have been made in different ways. One model uses gelatine methacryloyl/polyethylene (glycol) diacrylate hydrogels to create a scaffold that mimics the tumor microenvironment into which the A375 cells were seeded (Duan et al., 2022). Collagen 3D-printed scaffolds were used in another model and were shown to improve the maintenance and survival of cryopreserved patient-derived melanoma explants and retained melanoma biomarkers for 21 days (Jeong et al., 2021). Models such as this are a promising step towards producing physiologically and morphologically accurate pathological skin models which could help to improve understanding of conditions and to develop new medical treatments.

Collecting enough cells is a major challenge for pathological models. However, genome editing and induced pluripotent stem cells (iPS) are promising ways to obtain these cells. Unfortunately, there is currently not enough data to know whether iPS would revert to stem cells and create tumours if they were used in patients. It is also unknown if grafted cells can migrate around the body causing harm to the patient.

### 5.4 The ability to personalize skin models

Patient-specific skin substitutes using autologous cells can be made for personalized therapies. For example, personalized tumor or disease models may help establish patient-specific medications with personalized drug dose and composition (Aimar et al., 2019; Jamróz et al., 2018). This ability to adapt and personalize treatments to each patient will hugely impact personalized medicine development and could improve quality of life (Shopova et al., 2023; Serrano et al., 2023; Konta et al., 2017). Additionally, in the future, the development of multiple organs-on-a chip will enable a chemotherapy drug to be perfused through a personalized tumor model which is attached to a healthy tissue model to examine the effects the drug has on the tumor and healthy tissue (Biselli et al., 2017). Potentially, oncologists may be able to send a patient’s cancer cells to a laboratory along with the chemotherapy to test the effect on 3D models (Tibbetts, 2021). 3D bioprinting could be able to quickly and reliably produce biomimetic skin equivalents which meet both industrial and clinical needs for personalized treatments (Mahfouzi et al., 2021). In fact, combining induced pluripotent stem cells and 3D bioprinting could produce major advances in skin disease models which will improve the efficacy of developing and testing personalized treatments at lower costs (Gu et al., 2017; Li et al., 2018). Additionally, personalized tissue, such as skin grafts, are being researched which will minimize rejection risks thus improving tissue regeneration (Agarwal et al., 2020).

Bioprinting could revolutionize personalized medicine and has the potential to become the future gold standard for healthcare (Shopova et al., 2023).

### 5.5 The ability to control, standardize, and automate production

Manual fabrication has limited accuracy and consistency. Models made by different people or even the same person can vary due to pipetting variabilities. This is an issue as pharmaceutical screening and skin disease modelling need standardized models for reliable, accurate experiments. 3D bioprinting provides a versatile, easily adjustable, and modifiable automated process (Sarkiri et al., 2019). Reproducibility is also important. *In vitro* expansion of cells obtained from a single skin biopsy can produce vast numbers of cells enabling many skin models of the same origin to be created thus ensuring reproducibility (Haj-Ahmad et al., 2015). Additionally, with self-assembled manual fabrication models, the dermal equivalent is produced using fibroblasts which secrete their own ECM. The final dermal matrix is constructed through manual layering of these sheets which is a tedious, slow process and can take several weeks to produce sufficient matrix (Sarkiri et al., 2019; Jean et al., 2009; Roy et al., 2018). Even when robots replace humans, the process is the same as manual fabrication, so it remains time consuming. 3D bioprinting on the other hand can be a faster way to produce consistent and robust models (Vijayavenkataraman et al., 2018; Nguyen and Pentoney, 2017).

However, bioprinting systems are highly complex and require specialized personnel to maintain, sterilize and fix the machines. Additionally, the cost of printers, bioinks, cells, and maintenance remains high (Sarkiri et al., 2019; Vijayavenkataraman et al., 2018) and the printing process is longer than light-based printing. The required time and cost can therefore make scaling up difficult. Suppliers are working on new, faster technologies including light-based bioprinting or adding multiple heads to increase throughput.

Overall, compared with conventional techniques, 3D bioprinting enables high fidelity, high throughput, and time-saving testing and research, for example, with pharmaceutical and cosmetic screening or toxicity and allergenicity testing. Additionally, 3D bioprinting can tackle many of the limitations experienced with conventional tissue engineering. For example, with conventional tissue engineering, models are size restricted whereas with bioprinting, all shapes and sizes are possible and are only limited by the bed size. Also, conventional techniques are expertise dependent since the operator is trained to deposit cells correctly, but this step is automated with 3D bioprinting meaning it is not operator dependent, and variability is reduced. Furthermore, bioprinting is faster than conventional techniques but currently operators are still required to change plates. This will soon change due to the complete automation of the entire process.

## 6 Future perspectives

### 6.1 *In situ* bioprinting

*In situ* bioprinting is the next step in translating bioprinting technology from bench to bedside (Figure 3). This involves printing bioink directly onto the affected area to create or recover living skin tissue (Kang et al., 2022a; Hakimi et al., 2018). With this technology, customized constructs which perfectly match the skin defect can be made and *in situ* crosslinking improves mechanical strength. Automated *in situ* bioprinting rapidly fabricates a 3D scaffold with high accuracy and minimal invasion or errors. Handheld *in situ* bioprinting is a simplified, portable technique using extrusion bioprinting which is minimally invasive and easy to sterilize. It could have effective and rapid wound healing applications. The clinician can adjust the application according to the wound shape or patient movement. Pore-forming aqueous two-phase emulsion bioink used in a handheld bioprinter was shown to have good elasticity, withstand repeated mechanical compressions, and facilitate oxygen and liquid transport and cell proliferation and spreading (Ying et al., 2020). Fibrin incorporated into mesenchymal stem/stromal cell-laden hyaluronic acid hydrogel was found to increase epithelization speed and thickness and decrease scarring and contracture (Cheng et al., 2020). However, there is lower resolution, heterogeneous deposition, and reduced reconstruction precision. Robotic arm methods improve precision, and spatial deposition of the dressing and mobile skin bioprinting systems can rapidly manage extensive wounds (Kang et al., 2022a).

A challenge for these *in situ* bioprinted wound healing methods is an inadequate nutrient and oxygen supply. To overcome this, one study introduced oxygenic photosynthesis unicellular microalga (Chlorella pyrenoidosa) into the 3D scaffolds. These microalgae-laden scaffolds accelerated chronic diabetic wound closure by increasing angiogenesis, alleviating local hypoxia and promoting ECM synthesis (Wang et al., 2022).

Additionally, since hydrogels are highly hydrated, they are vulnerable to microbial infection. To overcome this, studies have been conducted using antibacterial polysaccharide composites crosslinked with gallium. These were active against Gram-negative and positive bacteria and particularly *S. aureus* and *P. aeruginosa* (Rastin et al., 2021). Antimicrobial drugs such as nafcillin have also been incorporated into bioink (Si et al., 2019).

While autografting remains the gold standard where possible, acellular substitutes exist including AlloDerm^®^, Integra^®^ and Biobrane^®^. There is also StrataGraft^®^ which is an FDA-approved bioengineered, allogeneic skin substitute to treat adults with thermal burns containing intact dermal elements (DPT burns). StrataGraft^®^ contains human keratinocytes and dermal fibroblasts which are grown on a collagen hydrogel matrix, forming a fully stratified epithelial layer that acts as a viable barrier.

Several limitations remain for clinical translation of skin models. It takes a long time to obtain a fully reconstructed skin model, and all protocols used must be compliant with clinical regulations. Currently, only healthy skin models could potentially be translated to clinical situations mainly using conventional methods but, a few clinical trials with bioprinting are ongoing (Deepa and Bhatt, 2025). Additionally, models cannot be sterilized like other medical devices, so they need to be produced in a completely sterile way. Tissue engineered skin substitutes are classified as class III medical devices in Europe and by the FDA meaning they present a high risk to patients and require the most stringent regulations and review processes making commercialization more complicated.

## 7 Conclusion

Despite pathological skin models created using conventional techniques having multiple uses, they remain limited by technical constraints which restrict size and prevent spatial organization from being accurately mimicked. Bioprinting techniques could overcome some of these limitations by enabling precise control over spatial organization to create complex, three-dimensional skin models. In terms of healthy skin models, these techniques are already revolutionizing personalized medicine, automating model production and supporting translational research. They could also represent a transformative approach for developing advanced pathological skin models which accurately mimic *in vivo* conditions. The limitations linked to bioprinting constitute research areas for future perspectives and opportunities, industrial scale up, accessibility to various biological materials through genetic manipulation and clinical applications.
